# Whole genome sequencing and genotyping *Klebsiella pneumoniae* multi-drug resistant hospital isolates from Western Kenya

**DOI:** 10.1099/acmi.0.000667.v4

**Published:** 2024-01-22

**Authors:** Victor Dinda, Andrew Nyerere Kimang’a, Daniel Kariuki, Anthony Wawire Sifuna, Thomas James O’Brien, Martin Welch, Oleg N. Reva

**Affiliations:** ^1^​ Department of Medical Laboratory Science, Masinde Muliro University of Science and Technology, Kakamega, Kenya; ^2^​ Department of Medical Microbiology, Jomo Kenyatta University of Agriculture and Technology, Nairobi, Kenya; ^3^​ Department of Biochemistry, Jomo Kenyatta University of Agriculture and Technology, Nairobi, Kenya; ^4^​ Department of Medical Biochemistry, Masinde Muliro University of Science and Technology, Kakamega, Kenya; ^5^​ Department of Biochemistry, University of Cambridge, Hopkins Building, Cambridge, UK; ^6^​ Centre for Bioinformatics and Computational Biology, Department of Biochemistry, Genetics and Microbiology, University of Pretoria, Pretoria, South Africa

**Keywords:** *Klebsiella pneumoniae*, environmental isolate, antibiotic resistance, whole genome sequence, MLST

## Abstract

**Objectives.:**

*Klebsiella pneumoniae* are a frequent cause of nosocomial infections worldwide. Sequence type 147 (ST147) has been reported as a major circulating high-risk lineage in many countries, and appears to be a formidable platform for the dissemination of antimicrobial resistance (AMR) determinants. However, the distribution of this pathogen in Western African hospitals has been scarcely studied. The main objective of this work was to perform whole genome sequencing of *K. pneumoniae* isolates from a referral hospital in Kakamega (Kenya) for genotyping and identification of AMR and virulence determinants.

**Methods.:**

In total, 15 *K*. *pneumoniae* isolates showing a broad spectrum antimicrobial resistance were selected for whole genome sequencing by Illumina HiSeq 2500 platform.

**Results.:**

ST147 was the dominant lineage among the highly-resistant *K. pneumoniae* isolates that we sequenced. ST147 was associated with both community- and the hospital-acquired infections, and with different infection sites, whereas other STs were predominantly uropathogens. Multiple antibiotic resistance and virulence determinants were detected in the genomes including extended-spectrum β-lactamases (ESBL) and carbapenemases. Many of these genes were plasmid-borne.

**Conclusions.:**

Our data suggest that the evolutionary success of ST147 may be linked with the acquisition of broad host-range plasmids, and their propensity to accrue AMR and virulence determinants. Although ST147 is a dominant lineage in many countries worldwide, it has not been previously reported as prevalent in Africa. Our data suggest an influx of new nosocomial pathogens with new virulence genes into African hospitals from other continents.

## Data Summary

*Klebsiella pneumoniae* strains were isolated from clinical specimens, identified by bacteriological methods and tested for antibiotic resistance. Selected strains showing a broad spectrum of antibiotic resistance were sent to MicrobesNG (Birmingham, UK) for extraction of genomic DNA and sequencing on the Illumina HiSeq 2500 platform. DNA reads were quality controlled and assembled using SPAdes version 3.14.0. Contig annotation was performed by Prokaryotic Genome Annotation Pipeline (PGAP V.6.6). Antibiotic resistance genes were predicted using the RGI/CARD web-service (https://card.mcmaster.ca/analyze/rgi). MLST typing was performed and virulence genes were identified using Kleborate software and the VFDB database. Whole genome sequences were deposited at NCBI under accession numbers shown in Table 1.

## Introduction

*Klebsiella pneumoniae* is emerging as a major clinical and public health threat, as is now reported to be responsible for up to one third of all Gram-negative infections. The organism is an opportunist and is becoming an increasingly prevalent source of nosocomial infections in the airways, urinary tract (where it ranks only second behind *Escherichia coli* as a causative agent) and surgical wound sites. *K. pneumoniae* is also a cause of serious community-acquired infections, especially pneumonias. The death rate for patients with *K. pneumoniae*-associated pneumonia is high, even following antibiotic treatment. Community-acquired infections appear to be linked with the spread of high-risk lineages such as ST147. Furthermore, clone ST147, is emerging globally as an important vehicle for the dissemination of AMR determinants [1]. In Low and Middle Income Countries (LMICs), multi-drug resistance (MDR) among clinically-significant Gram-negative bacteria is increasingly becoming a cause of increased morbidity, accounting for an estimated 40 % of mortality [2].

Although community- and hospital-acquired transmission pathways for *K. pneumoniae* have clearly been demonstrated in high income countries [3], there is generally paucity of such evidenced studies from sub-Saharan Africa. Because of the absence of such data, policy makers and implementors therefore have limited evidence to rely on when allocating resources towards infection, prevention and control (IPC) programmes. In the current study, we attempt to rectify this and decipher the possible pathways that may have been involved in a sudden surge in *K. pneumoniae* occurrence in a regional referral health facility in Western Kenya during the period December 2015 to May 2016.

While monitoring for extended spectrum β-lactam resistance among *Enterobacteriaceae* isolates obtained from Kakamega County General Teaching and Referral Hospital (KCGTRH) in Western Kenya, we noticed an increase in the frequency of multi-drug resistant *K. pneumoniae*. These isolates were resistant to a broad range of structurally distinct classes of antimicrobial agent. We therefore used whole genome sequencing (WGS) to analyse the genetic structure of a selection of these *K. pneumoniae* isolates. Moreover, by analysing the encoded virulent traits, antibiotic resistance genes, and plasmid sequences in each isolate, we provide an evidence-based description of the local species diversity associated with the outbreak, as well as possible transmission pathways.

Our study strongly suggests that antibiotic resistance is acquired in the *K. pneumoniae* lineages through acquisition of virulence plasmids that are enriched with antibiotic/antimicrobial resistance genes (ARGs). We show that WGS is a powerful, and increasingly economical tool in the fight against communicable infections in the LMIC healthcare envioronment, and can even potentially discriminate between the lineages that cause community-associated infections and nosocomial infections. Such data should be invaluable for health authorities in LMIC, enabling them to identify key areas for intervention and resource deployment.

## Methods

### Bacterial isolation and identification

Samples were obtained from KCGTRH (Kenya) over the period from December 2015 to May 2016. *K. pneumoniae* were recovered from patients seeking treatment for various ailments including respiratory tract infections, urinary tract infections (UTIs), sepsis and wounds. Infections were designated as hospital- or community-acquired to distinguish between different possible sources of infection. The nosocomial infections were defined as those acquired by patients after hospitalization, manifesting 48 h after admission. For the urine specimens, midstream urine samples were collected and cultured on MacConkey agar (Hi-Media, India). Wound samples were collected by swabbing the wound, and were also cultured on MacConkey agar (Hi-Media, India). For blood culture, two sets of sterile draws (aerobic and anaerobic) from two separate venipuncture sites were drawn and the samples were then incubated in a blood culture incubator (BACTEC) at 35–37 °C for 24–36 h. The blood samples were then sub-cultured inoculated by streaking on blood agar base BAP, Chocolate blood agar, MacConkey and Sabouraud Dextrose Agar (SDA) for further identification. Colony-pure isolates were confirmed as *K. pneumoniae* using API20E (BioMérieux) biochemical test strips following the manufacturer’s instructions.

### Antimicrobial susceptibility testing

A total of 27 *K*. *pneumoniae* isolates were subjected to antibiotic susceptibility profiling using the Kirby Bauer disc diffusion method. We tested amikacin, amoxicillin/clavulanate, ampicillin/sulbactam, cefepime, cefotaxime, ceftazidime, ceftriaxone, cefuroxime, gentamicin, imipenem, meropenem, nitrofurantoin, piperacillin/tazobactam, ciprofloxacin, and trimethoprim/sulfamethoxazole. *Escherichia coli* ATCC 25922 was used as a reference as per Clinical and Laboratory Standards Institute (CLSI) guidelines (2017). Of the 27 isolates tested, nine were multi-drug resistant. For long-term storage, cultures of the *K. pneumoniae* isolates were frozen at −80 °C in trypticase soy broth supplemented with 15 % v/v glycerol.

### Genomic DNA sequencing

Whole genome sequencing was carried out by MicrobesNG (Birmingham, UK) using an Illumina HiSeq 2500 platform. Briefly, a single colony of each strain was picked and suspended in 100 µl of sterile 1×phosphate-buffered saline (PBS) (Oxoid, UK). The suspension was spread thickly (using a sterile loop) onto a fresh LB-agar plate and incubated at 37 °C overnight. Dense colony growth was then scraped off and sent to MicrobesNG in supplied bar-coded bead tubes. Sequencing (30-fold depth) was carried out using an Illumina HiSeq 2500 platform, with 2×250 bp paired-end reads. The reads were trimmed using Trimmomatic v0.30 with a sliding window quality cut-off of Q15. The *de novo* assembly of contigs was done using SPAdes version 3.14.0 with default settings. The resulting contigs were scaffoled by alignment to the closest reference sequences found in NCBI by MegaBLAST search. The closest reference genomes are listed in Table 1. Gaps between contigs were patched by the respective genomic fragments from the reference genomes. Then the original DNA reads were mapped against the resulting genome sequences by the Bowtie2 algorithm implemented in Unipro UGENE v48.1 [4] to verify the patched regions and generate consensus sequences. Preliminary genome annotation was done using the RAST Annotation Server [5] with automatic fix error function, which checks and fixes possible sequencing and genome assembly errors. The NCBI Prokaryotic Genome Annotation Pipeline (PGAP v.6.6) [6] was later used to refine the annotation. BioPython v1.81 was used to find *dnaA* coding sequences in the annotated genomes and to rearrange the sequences in a way such that each sequence starts 400 bp upstream of *dnaA* in the leading DNA strand (i.e. to correspond to the chromosomal replication origin). The genomic sequences were finalized by another round of mapping the original DNA reads against the genome sequences to confirm circularity of the chromosomes and to verify modifications introduced by the RAST fix error function. Quality of the resulting consensus sequences was controlled by the programme CheckM2 v1.0.2 [7]. Plasmid contigs were identified using mlplasmids 1.0.0 [8] and the *mob_recon* function of the Mob-Suite v3.1.7 utility (https://github.com/phac-nml/mob-suite) [9] with the default parameter setting. Further analysis and genotyping of the plasmid sequences was performed by using function *mob_typer* of Mob-Suite. Whole genome sequences were deposited at NCBI under the BioSample accession numbers shown in Table 1.

**Table 1.** Accession numbers of *K. pneumoniae* genome sequences at GenBank NCBI

Strain no.	BioSample	CheckM2 metrics (Completeness / contamination)	Reference genomes
CK1	SAMN24338476	100 % / 0.76 %	CP054990.1
CK2	SAMN31566495	99 % / 0.2 %	CP035196.1
CK3	SAMN31566649	100 % / 0.65 %	CP044047.1
CK4	SAMN24369220	100 % / 0.54 %	CP023839.1
CK5	SAMN24369239	99.9 % / 0.95 %	CP050371.1
CK6	SAMN24369281	100 % / 1.52 %	CP024916.1
CK7	SAMN24369410	100 % / 1.52 %	CP024916.1
CK8	SAMN24370162	100 % / 1.12 %	CP044047.1
CK9	SAMN24371033	100 % / 1.52 %	CP024916.1
K10	SAMN24371047	100 % / 1.1 %	CP052224.1
K11	SAMN24377653	100 % / 1.1 %	CP052224.1
K12	SAMN24377668	100 % / 1.1 %	CP052224.1
K13	SAMN24386524	100 % / 1.1 %	CP052286.1
K14	SAMN24386561	100 % / 0.08 %	CP043859.1
K15	SAMN24386923	100 % / 1.1 %	CP030315.1

### Gene ortholog prediction and phylogenetic inferencing

Clusters of orthologous groups (COGs) in sequenced genomes were predicted using the programme OrthoFinder [10] with default parameters. The sequences of each COG were aligned using the muscle v3.6 algorithm with the parameters set by default [11]. Ambiguous parts of the alignments were removed using the programme Gblocks v0.91 [12] with the default parameter settings. COG alignments were concatenated using BioPython scripts into superstring alignments for further phylogenetic inferences using the Neighbour-Joining algorithm implemented mega-X [13]. Alignment of plasmid sequences was performed by Mauve 20150226 [14]. Here, the sequence similarity is the ratio of base pairs shared between two genomes to their average genome length. This similarity estimate is then converted to a distance value for the NJ distance matrix. The progressiveMauve algorithm produces a dendrogram of sequence similarity relations stored in an alignment.guide_tree file. The dendrogram was visualized by mega-X.

### Genome typing, virulence and antibiotic resistance prediction

Species confirmation of the sequenced microorganisms, multilocus sequence typing (MLST), capsule typing, antibiotic resistance and virulence predictions were done using Kleborate v2.3.2 (https://github.com/klebgenomics/Kleborate) [15] and Kaptive v2.0.7 (https://github.com/klebgenomics/Kaptive) [16]. MLST predictions were controlled by searching through the BIGSdb (https://bigsdb.pasteur.fr/) and CBS databases [17, 18]. Antibiotic resistance and virulence genes additionally were predicted using Resistance Gene Identifier (RGI 6.0.3) with the CARD database (https://card.mcmaster.ca/analyze/rgi) [19] and VFDB database records for *Klebsiella* [20]. The Kleborate and Kaptive predictions were combined with the phylogenetic tree and visualized using the Microreact Web-service (https://microreact.org/). Mobile horizontally-acquired genomic islands (GIs) were identified using SeqWord Genomic Island Sniffer (http://seqword.bi.up.ac.za/sniffer/index.html) [21]. SeqWord identifies horizontally-acquired genomic elements by analysing patterns of oligonucleotide signatures.

## Results

### Antibiotic susceptibility profiles of the selected *Klebsiella pneumoniae* isolates

Hospital- and community-acquired *Klebsiella* pathogens were isolated from a range of infection types and sites over a ca. 6 month period, as outlined in the Methods. Each isolate was tested for its ability to grow on a selection of structurally-diverse antibiotics with different targets and different modes of action. The antibiotics tested included β-lactams, cephalosporins and β-lactamase inhibitors (amoxicillin/clavulanate (AMC), piperacillin/tazobactam (TZP), ceftazidime (CAZ), meropenem (MEM), cefotaxime (CTX), cefuroxime (CXM), ceftriaxone (CRO), cefepime (FEP), ampicillin/sulbactam (SAM) and imipenem (IMP), aminoglycosides (gentamicin [GM] and amikacin [AMK]), a DNA gyrase inhibitor (ciprofloxacin, CIP), folate metabolism inhibitors (trimethoprim/sulfamethoxazole, SXT), and nitrofurantoin (NIT), which is known to be a very effective antibiotic with a poorly-defined mode of action. Of the 27 isolates we tested, nine were resistant to all of these antibiotics. The genome of each of these super-resistant isolates was sequenced. Some isolates were resistant to all of the tested antibiotics except one; for example, MEM (isolate CK1), IMP (isolate CK3), SXT (isolate CK4), NIT (isolate CK5) and AMK (isolate K15). We also sequenced the genome of each of these isolates too. Finally, isolate CK2 was also included in the genome sequencing, since it was sensitive to all of the antibiotics tested except SAM. With the exception of CK1, all of the isolates subjected to whole genome sequencing were harvested in the period March 2016–April 2016. Genomic DNA was collected from single colonies of the sampled *K. pneumoniae* strains following overnight growth on LB-agar at 37 °C. DNA samples were sequenced using an Illumina HiSeq 2500 platform with 2×250 bp paired-end reads, and assembled using SPAdes v3.14.0. The final genome assemblies were annotated by PGAP. Genome completeness and purity were confirmed by CheckM2 analysis (Table 1). All of the isolates were unambiguously identified as *Klebsiella pneumoniae* following analysis using of the whole genome sequences using Kleborate (Table S2, available in the online version of this article).

### Epidemiology and typing of *Klebsiella pneumoniae* isolates

**Fig. 1.**
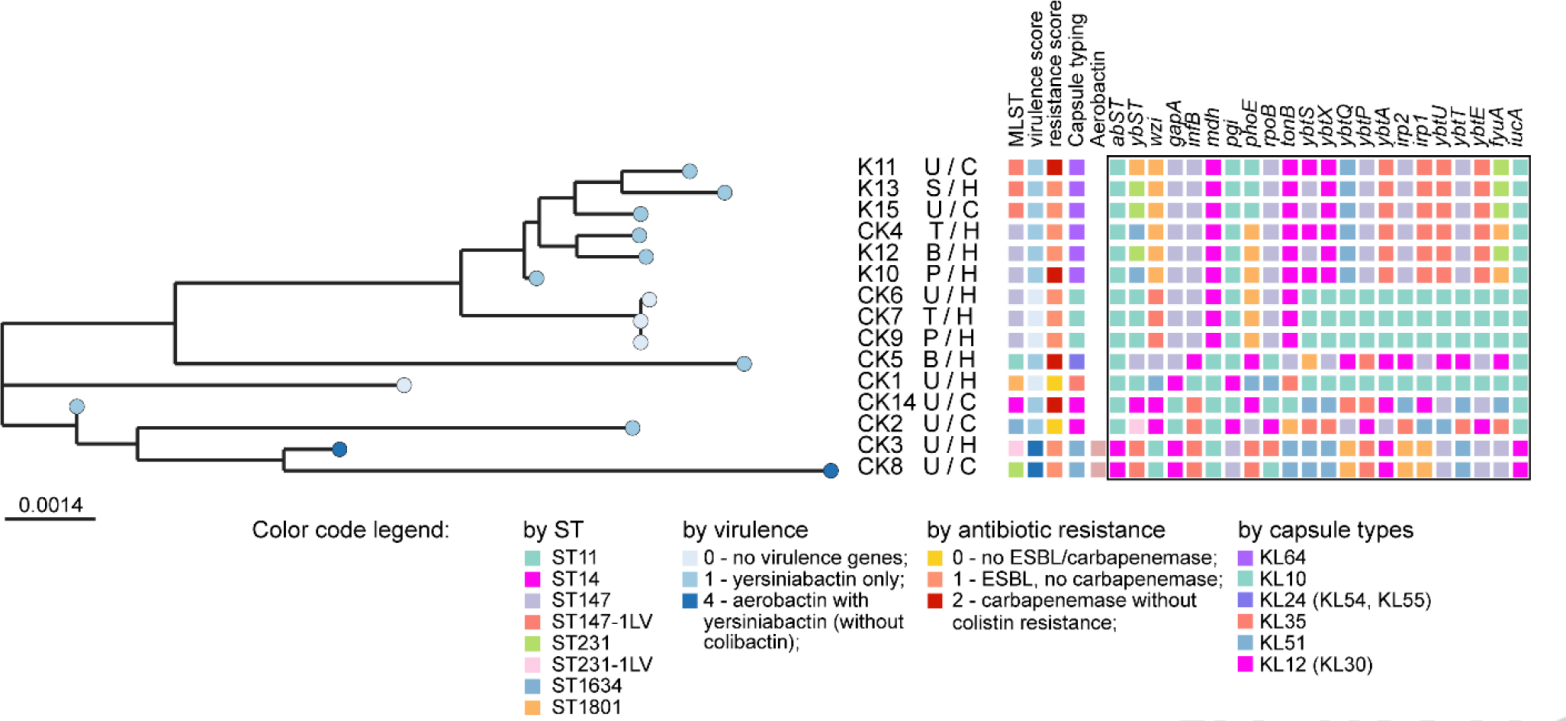
Phylogeny and genotypes of the *K. pneumoniae* isolates shown in the Neighbour-Joining tree as nodes shaded in accordance with Kleborate predicted virulence scores. Predicted MLST types of the isolates (ST), extracellular capsule types, virulence and antibiotic resistance categories are depicted by colour as explained in the figure legend. Sequence variants of marker genes traditionally used for MLST are depicted in the framed box. Infection sites and infection acquisition were abbreviated: Pus (**p**); Blood (**b**); Urine (**u**); Sputum (**s**); Tracheal aspirate (**t**); Hospital-acquired (**h**); Community-acquired (**c**).

The obtained genome sequences were used for MLST typing of the isolates. Nine out of the 15 isolates were assigned to ST147 (including a sub-type ST147-1LV, where 1LV indicates that an additional SNP is present compared with the previously published ST). The ST147 isolates were split in the phylogenetic tree into several clusters characterized by two different polysaccharide capsule types, KL64 and KL10. Two isolates CK3 and CK8 belonged to ST231 and ST231-1LV (respectively) but had the same capsule type, KL51. The remaining four minor isolates were assigned to ST11, ST14, ST1634 and ST1801, and were characterized as having three different capsule types (Fig. 1). Additional information about sources and dates of isolation of the clinical isolates, and their respective STs, are given in Table 2.

**Table 2.** Source and MLST typing of the sequenced *K. pneumoniae* isolates

**Isolate identity**	**Sequence type (ST)**	**Sample site**	**Type of hospital care**	**Date of isolation**	**Age**	**Sex**	**Hospital (H)/Community (C) aquired**
CK1	1801	U	IP	28 December 2015	42	M	H
CK2	1634	U	OP	21 March 2016	66	F	C
CK3	231	U	IP*	07 March 2016	36	F	H
CK8		U	OP	01 April 2016	62	F	C
CK5	11	B	IP	11 March 2016	39	F	H
CK14	14	U	OP	18 March 2016	36	M	C
CK4	147	T	IP/ICU*	25 April 2016	59	F	H
CK6		U	IP/ICU	25 April 2016	48	M	H
CK7		T	IP	07 May 2016	72	F	H
CK9		P	IP	05 April 2016	28	M	H
K10		P	IP*	21 March 2016	56	M	H
K11		U	OP	30 March 2016	49	F	C
K12		B	IP/ICU*	07 April 2016	57	F	H
K13		S	IP*	31 March 2016	11	F	H
K15		U	OP	11 March 2016	47	F	C

B, Blood; F, Female; IP*, In-patient, but admitted for <48 h; IP, In-patient; IP/ICU, In-patient admitted in intensive care; M, Male; OP, Out patient; P, Pus; S, Sputum ; T, Tracheal aspirate; U, Urine.

### Phylogenetic relations between *Klebsiella pneumoniae* isolates

DNA reads generated by the Illumina sequencer were assembled using SPAdes into contigs, which then were scaffolded and joined to yield whole genome sequences. The genomes were annotated using the NCBI genome annotation robot PGAP. Following this, clusters of orthologous protein-coding genes (COG) were identified, translated to amino acid sequences, and aligned. Alignments of protein sequences of all COGs (2637 orthologous protein-coding genes) were concatenated into a superstring alignment comprising 805 256 positions (inclusive of amino acid residues and gaps). A Neighbour-Joining phylogenetic tree was then generated based on this alignment. The genetic typing by MLST is in good agreement with the phylogenetic relations inferred between the isolates based on the NJ tree of the superstring alignment (Fig. 1). The phylogenetic tree structure was consistent with the genomic MLST typing. The isolates of ST147 were associated with both hospital-acquired (HA) and community-acquired (CA) infections, and with a variety of infection sites. By contrast, isolates CK1, CK2, CK3, CK8, and CK14 all belonged to different STs, but were all associated with urinary tract infections.

### Identification of antibiotic resistance genes and their distribution between chromosomes, genomic islands and plasmids

Numerous ARG and virulence determinants were identified in the isolates using the public databases RGI-CARD, VFDB and Kleborate software (Table S1). These included various efflux pumps (*acrBDFS*, *emrABKRY*, *oqxAB*, *tet(E*), *mdfABCEFNOP*) and their known regulators (*baeR*, *crp*, *evgA* and *gadX*), as well as a selection of other important regulators that have been previously associated with antibiotic resistance (*baeSR*, *evgAS* and *kdpE*). We also confirmed the presence of certain stress response genes (*cpxA*, *marA* and *tolC*) that have been implicated in modulating drug efflux under some conditions [22, 23]. Three β-lactamases were identified; the sulfhydryl reagent-variable β-lactamase SHV-52, the extended spectrum β-lactamase CTX-M-130, and the AmpC-family enzyme [24], FOX-1. Additional AMR-associated genes included the MFS-family drug transporters, *mdtH* and *mdtM*, and an SMR-type efflux pump (*kpnEF*). Moreover, several genes involved in modifying the bacterial cell envelope in response to stress were identified, including genes involved in lipid A biosynthesis (*pmrF* and *ugd*) and modification (*eptA*) [25].

Other AMR-associated genes were also present in the isolates. These included *fosA5* (a glutathione *S*-transferase implicated in fosfomycin resistance), *sul3* (implicated in sulfonamide resistance), chloramphenicol exporters (*cmlB* and *floR*) and chloramphenicol acetyltransferases (*catI* and *catB11*), tetracycline resistance genes (including *tet*(*32*) which confers ribosomal protection against tetracycline, and the tigecycline resistance gene, *tet(X4*)), a phosphotransferase (*mphB*) that phosphorylates macrolide antibiotics, the bacitracin phosphorylase *bacA*, and the microcin exporter *yojI*.

Many of the above mentioned ARGs were distributed across the core parts of the chromosomes (conserved across the isolates) and various mobile elements. For example, *acrE*, *evgA*, *evgS* and *ugd* were found within predicted horizontally-acquired genomic islands (GIs) in all the isolates. By contrast, many of the other AMR determinants exhibited a less homogenous distribution between isolates. For example, the β-lactamase CTX-M-130, fosfomycin resistance gene *fosA5*, chloramphenicol exporter *cmlB*, efflux pump regulator *acrS*, MFS transporter *mdtM*, tetracycline resistance gene *tet32*, inner membrane transporter *acrFE*, efflux pumps *oqxAB* and *mdfA*, antibiotic resistance genes *yojI* and *floR*, and the multi-drug transporter *ymrKY* were each found within GIs in at least one of the selected isolates. The numbers of ARG associated with GIs in the selected microorganisms varied from three (in CK14) to seven (in CK5 and CK8).

An overall prediction of the virulence and antibiotic resistance status of the isolates was performed using Kleborate (Fig. 1 and Table S2). Two strains, CK3 and CK8 (both ST231) isolated from the hospital and from the community (respectively), were predicted to encode both the aerobactin and yersiniabactin iron acquisition systems. These siderophore systems contribute significantly to the virulence of *Klebsiella* and *Escherichia* spp. [26–28]. Another nine strains possessed the yersiniabactin system alone, and four of the isolates encoded neither siderophore system. All strains except for two (CK1 and CK2) encoded extended-spectrum β-lactamases (ESBL, CTX-M-15) [29] and Kleborate predicted carbapenemases in four strains (OXA-181 in strains K10, K11 and CK14; and NDM-1 in CK5) rendering resistance to carbapenem antibiotics [30]. The streptomycin resistance genes, *strAB*, and the sulfonamide resistant dihydropteroate synthase, *sul2*, were found in CK5, K10, K11, K12, K13, and K15. Strain K14 also possessed an aminoglycoside-modifying enzyme, encoded by *sat*-2. Mutations in *gyrA* and *parC* associated with fluoroquinolone resistance [31] were identified in all strains except for CK1 and CK2. Finally, CK3, CK4, CK8, K10, K11, K12, K13, K14 and K15 all carried mutations in the outer membrane porins, *ompK35* and *ompK36*, associated with doripenem-colistin resistance [32].

**Fig. 2.**
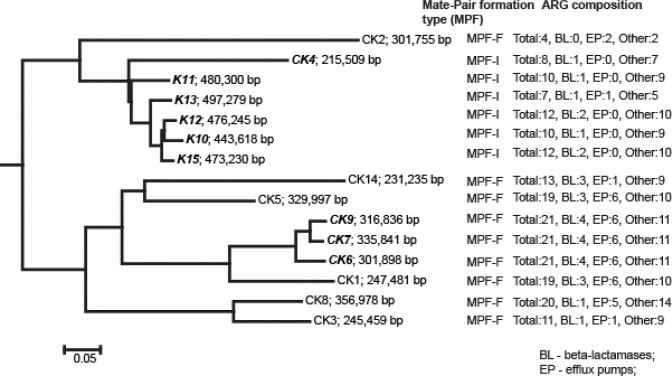
Dendrogram showing sequence similarity of the plasmids found in the *K. pneumoniae* isolates. The tree was rooted at the average branch location not representing the common ancestry. The total numbers of antimicrobial resistance genes (ARG) and the numbers of β-lactamases (BL), efflux pumps (EP) and other categories of ARG are shown on the right-hand side of the figure.

Plasmid-associated contigs were identified using mlplasmid (a support vetor machine model trained on *K. pneumoniae*). Alignment of plasmid contigs against each other using the programme Mauve (Fig. S1) allowed us to construct the dendrogram in Fig. 2. A search for similar plasmids in the NCBI was perfomed using Mob-Suite software [9]. All the plasmids were predicted by this tool as conjugative broad-range plasmids belonging to mate-pair formation (MPF) types I and F. The MPF classification of the plasmids is in agreement with their clustering in the dendrogram (Fig. 2). The full list of determined Mob-Suite metrics is shown in Table S3. In parallel, we also screened for plasmid-borne ARGs. A number of plasmid-associated ARGs were identified, including β-lactamases (BL) and efflux pumps (EP) (Fig. 2). Interestingly, many of these ARGs were paralogues of genes already present on the chromosomes. This may indicate that plasmid-borne ARGs (and possibly, also those associated with other horizontally-acquired GIs) may confer a greater degree of antibiotic resistance than the homologous genes located in the core genome. Alternatively, the elevated copy number of the duplicated genes may be responsible (i.e. a gene dosage effect). Detailed information on the specific plasmid-borne ARGs associated with each strain is provided in Supplementary Table S2.

Alignment of the plasmid contigs against one another revealed some level of sequence similarity, with the greatest identity (> 90 %) for the plasmids from the ST147 strains K10, K12, K13 and K15, all of which belong to ST147. We also note that the hospital-acquired ST147 strains CK6, CK7 and CK9 all appear to share common plasmids.

However, this similarity between plasmids only to some extent corresponded with the phylogenetic relationship between the isolates (cf. Fig. 1 versus Fig. 2). Two types of plasmid were associated with the isolates of ST147. Isolates K10, K12, K13 and K15 contain large plasmids of 440–490 kb size. Assuming that the evolution of virulence plasmids in the antibiotic era is towards rendering multidrug resistance, these plasmids may have evolved from the smaller, ancestral plasmids represented by those in isolates CK2 (ST1634) and CK4 (ST147) through the acquisition of additional ARGs. The ST147 isolates CK6, CK7 and CK9 contain another type of plasmid (size range 301–335 kb). These plasmids may also have evolved from smaller plasmids, although here, the ancestor appears to be similar to the plasmid in isolate CK1 (ST1801). This evolutionary pathway too is associated with an increase in the number of ARGs; indeed, the plasmids from CK6, CK7 and CK9 contain the largest number of plasmid-born ARG seen in the current study, even outnumbering the ARGs present in the much larger plasmids from isolates K10, K12, K13 and K15 (Fig. 2). The plasmid from the antibiotic-susceptible isolate, CK2, carried the smallest number of ARGs.

## Discussion

Our data indicate that ST147 was enriched among the highly-resistant *K. pneumoniae* isolates that we selected for whole genome sequencing. Indeed, of the 15 isolates that we analysed this way, nine were ST147. On the other hand, according to the Kleborate predictions (Fig. 1), the most worrisome isolates were the two strains of ST231.

To date, *K. pneumoniae* ST147 has been reported as a causative agent of high-risk hospital infections around the world, but mostly in the northern hemisphere [33–36]. However, at the time of publication, KCGTRH receives 5–10 patients per day with *K. pneumoniae*-associated infections. Our data indicate that (unless KCGTRH is somehow unusual) ST147 is now common among the hospital isolates from Western Kenya. By contrast, other recent studies did not observe *K. pneumoniae* ST147 in African hospitals [37–39], and *K. pneumoniae* lineages have been reported to be diverse and dominated by ST131, ST335, ST1193, ST10, ST14, ST15 and ST307. Indeed, only one paper has mentioned multi-drug resistant isolates of *K. pneumoniae* ST147 from Kenya [38]. However, although that paper was published in 2022, we note that sampling of the isolates analysed therein happened between 2015–2020. Given the dominance of ST147 observed in the current study, this may suggest that ST147 emerged in the region around that time. The significant shift in population structure of hospital-associated *K. pneumoniae* from the diverse lineages in [37] and [38] to the dominance of ST147 reported here is curious. One possibility is that the shift is linked with human migration, or by the abuse of antibiotics in the region (we recall here the plasmid-borne accrual of AMR and virulence genes in the ST147 isolates). Another possible factor may be that this lineage is not confined by an infection site. Consistent with this, there was no obvious correlation between the prevalence of ST147 and the type of infection (wound exudate, blood, tracheal aspirate, expectorated sputum, urinary). Moreover, we note that ST147 strains could equally be isolated either from hospitals or from the community [38]. Finally, another formal possibility is that discrepencies in the reported frequency of ST147 simply reflect a somewhat uneven geographical distribution of the lineage between clinics, or a recent influx of the lineage from other parts of the world. If so, this is of major concern, especially given that ST147 provides a formidable platform for the dissemination of AMR. At the very least, a comprehensive effort will be needed to understand the true distribution of this clone-type in the east African region.

Antibiotic resistance genes were identified in both the core genome and in genomic islands of all the isolates we examined in this study. We also identified many paralogous resistance determinants in the plasmidome of each isolate. The evolutionary success of ST147 appears to be associated with the acquisition of two broad-range plasmids, each enriched in ARGs. The proclivity of *K. pneumoniae* lineages to acquire plasmids from one another is well known [33]. However, AMR may not be the only determinant of ST success in the region. ST231 was also represented in our dataset, and worryingly, not only manifested multi-drug resistance, but was also enriched in virulence determinants as well as ARGs. These features were encoded by-and-large, on horizontally-acquired GI insertions in the ST231 chromosomes. Isolates of ST231 from China, Türkiye and Africa have previously been characterized and found to be associated with broad spectrum antibiotic resistance, especially against carbapenem antibiotics [40, 41].

## Supplementary Data

Supplementary material 1
