# Supplementary material for: Whole genome sequencing and genotyping Klebsiella pneumoniae multi-drug resistant hospital isolates from Western Kenya
Source: Access Microbiol. 2024 Jan 22;6(1):000667.v4. doi: 10.1099/acmi.0.000667.v4 (PMC10866029; doi:10.1099/acmi.0.000667.v4)
Supplement: Supplementary material 1 [file acmi-6-667.v4-s001.pdf]

Supplementary Table S1. Plasmid born antibiotic resistance genes (ARG).

K. pneumoniae isolates															Gene	Anntotation
CK1	CK2	CK3	CK4	CK5	CK6	CK7	CK8	CK9	CK10	K11	K12	K13	K14	K15		
+	--	--	--	--	+	+	--	+	--	--	--	--	+	--	*AAC(3)-IIc*	Aminoglycoside acetyltransferase
+	--	--	+	+	+	+	+	+	+	+	+	+	+	+	*AAC(6')-Ib*	Aminoglycoside acetyltransferase
--	--	+	--	+	--	--	--	--	--	--	--	--	--	--	*AAC(6')-Ib-cr*	Aminoglycoside acetyltransferase
--	--	+	--	--	--	--	+	--	--	--	--	--	--	--	*aadA12*	Aminoglycoside nucleotidyltransferase
+	--	--	--	+	+	+	--	+	+	+	+	+	--	+	*APH(3')-IIa*	Aminoglycoside phosphotransferase
+	--	--	--	+	+	+	--	+	+	+	+	+	--	+	*APH(6)-Ic*	Aminoglycoside phosphotransferase
--	--	--	+	--	--	--	+	--	+	+	+	+	+	+	*arr-8*	Ribosyltransferase
+	--	--	--	+	+	+	+	+	--	--	--	--	--	--	*baeR*	Response regulator that promotes the expression of MdtABC and AcrD efflux complexes
+	--	--	+	+	+	+	+	+	+	+	+	+	+	+	*catB11*	Chloramphenicol acetyltransferase
--	--	+	--	+	--	--	+	--	--	--	--	--	--	--	*catI*	Chloramphenicol acetyltransferases
--	--	--	--	--	--	--	--	--	--	--	--	--	+	--	*cmlB*	Chloramphenicol exporter
+	--	--	--	+	+	+	+	+	--	--	--	--	--	--	*cpxA*	Membrane-localized sensor kinase that is activated by envelope stress and promotes efflux complex expression
+	--	--	--	--	--	--	--	--	--	--	--	--	--	--	*CTX-M-130*	Beta-lactamase
--	--	+	--	--	--	--	+	--	--	--	--	--	--	--	*dfrA13*	Dihydrofolate reductase
--	--	+	--	--	--	--	+	--	--	--	+	+	+	+	*emrE*	Ethidium bromide and erythromycin efflux transporter
--	--	--	--	--	--	--	--	--	--	--	--	--	+	--	*emrR*	Negative regulator of multidrug efflux pumps
--	--	--	--	--	--	--	--	--	--	--	--	--	+	--	*ereA*	Erythromycin esterase
+	--	--	--	--	+	+	--	+	--	--	--	--	--	--	*evgS*	Sensor regulatory protein involved in drug resistance
--	--	+	--	--	--	--	+	--	--	--	--	--	--	--	*floR*	Chloramphenicol exporter
--	--	--	--	--	+	+	+	+	+	+	+	+	--	+	*H-NS*	Histone-like protein involved in global gene regulation including RND-type multidrug exporters.
+	--	--	--	+	+	+	--	+	--	--	--	--	--	--	*kdpE*	Transcriptional activator involved in the virulence and intracellular survival of pathogenic bacteria
--	--	+	--	--	--	--	+	--	--	--	--	--	--	--	*marA*	Global activator protein inducing MDR efflux pump AcrAB and porin OmpF
+	+	--	--	+	+	+	+	+	--	--	--	--	--	--	*mdtA*	Membrane fusion protein of the multidrug efflux complex
+	+	--	--	+	+	+	+	+	--	--	--	--	--	--	*mdtF*	Multidrug inner membrane transporter for the MdtEF-TolC efflux complex
--	--	--	+	--	--	--	--	--	--	--	--	+	--	--	*mdtG*	Major facilitator superfamily of transporters increasing fosfomycin and deoxycholate resistance
--	+	--	--	--	--	--	--	--	--	--	--	--	--	--	*mdtM*	Multidrug resistance protein
+	--	--	--	+	+	+	--	+	--	--	--	--	--	--	*mdtP*	Multidrug resistance efflux pump
--	--	+	+	+	--	--	+	--	+	+	+	+	+	+	*mphA*	Resistance enzymewhich preferentially inactivate 14-membered macrolides
+	+	+	--	--	+	+	+	+	--	--	--	--	--	--	*msbA*	Multidrug resistance transporter
--	--	--	--	+	+	+	--	+	--	--	--	--	--	--	*NDM-6*	Beta-lactamase
+	--	--	+	+	+	+	+	+	--	--	+	+	+	+	*OXA-232*	Beta-lactamase
--	--	--	--	--	--	--	--	--	--	--	--	--	+	--	*OXA-9*	Beta-lactamase
+	--	--	+	+	+	+	+	+	--	--	--	--	--	--	*qnrS8*	Quinolone resistance protein
--	--	--	+	--	--	--	+	--	+	+	+	+	--	+	*rmtH*	16s Ribosomal RNA methyltransferase that confers high resistance to all aminoglycosides
+	--	+	--	+	+	+	--	+	+	+	+	+	+	+	*SHV-52*	Beta-lactamase
+	--	+	--	+	+	+	+	+	+	+	+	+	+	+	*sul3*	Sulfonamide resistant dihydropteroate synthase
--	--	--	--	--	+	+	--	+	--	--	--	--	--	--	*TEM-126*	Beta-lactamase
+	--	--	--	+	+	+	--	+	--	--	--	--	--	--	*tet(E)*	Tetracycline efflux pump
19	4	11	8	19	21	21	20	21	10	10	12	13	13	12	**Total DRG**	

Supplementary Table S2. Output of analysis of *K. pneumoniae* genomes by Kleborate software

Metrics*	*Klebsiella pneumoniae* strains														
	CK1	CK2	CK3	CK4	CK5	CK6	CK7	CK8	CK9	K10	K11	K12	K13	K14	K15
species_match	strong	strong	strong	strong	strong	strong	strong	strong	strong	strong	strong	strong	strong	strong	strong
contig_count	1	1	1	1	1	1	1	1	1	1	1	1	1	1	1
N50	5463570	5409351	5483106	5502003	5495443	5568378	5580866	5332283	5570665	5753154	5664041	5719068	5717081	5519244	5718783
largest_contig	5463570	5409351	5483106	5502003	5495443	5568378	5580866	5332283	5570665	5753154	5664041	5719068	5717081	5519244	5718783
total_size	5463570	5409351	5483106	5502003	5495443	5568378	5580866	5332283	5570665	5753154	5664041	5719068	5717081	5519244	5718783
ambiguous_bases	yes (1800)	yes (5000)	yes (3400)	yes (5800)	yes (3700)	yes (3400)	yes (3500)	yes (9200)	yes (3500)	yes (3600)	yes (4800)	yes (4900)	yes (4100)	yes (4200)	yes (5300)
QC_warnings	ambiguous_bases	ambiguous_bases	ambiguous_bases	ambiguous_bases	ambiguous_bases	ambiguous_bases	ambiguous_bases	ambiguous_bases	ambiguous_bases	ambiguous_bases	ambiguous_bases	ambiguous_bases	ambiguous_bases	ambiguous_bases	ambiguous_bases
ST	ST1801	ST1634	ST231-1LV	ST147	ST11	ST147	ST147	ST231	ST147	ST147	ST147-1LV	ST147	ST147-1LV	ST14	ST147-1LV
virulence_score	0	1	4	1	1	0	0	4	0	1	1	1	1	1	1
resistance_score	0	0	1	1	2	1	1	1	1	2	2	1	1	2	1
num_resistance_classes	2	0	2	2	5	2	2	2	2	6	6	5	5	5	5
num_resistance_genes	2	0	1	1	5	1	1	1	1	8	8	7	7	4	7
Yersiniabactin	-	ybt 4; plasmid	ybt 14; ICEKp5	ybt 10; ICEKp4	ybt 15; ICEKp11	-	-	ybt 14; ICEKp5	-	ybt 10; ICEKp4	ybt 10; ICEKp4	ybt 10; ICEKp4 (truncated)	ybt 10; ICEKp4 (truncated)	ybt 14; ICEKp5	ybt 10; ICEKp4 (truncated)
YbST	0	65-1LV	376	384	230-2LV	0	0	376	0	384	384-1LV	384-2LV	384-2LV	140	384-2LV
Colibactin	-	-	-	-	-	-	-	-	-	-	-	-	-	-	-
CbST	0	0	0	0	0	0	0	0	0	0	0	0	0	0	0
Aerobactin	-	-	iuc 5	-	-	-	-	iuc 5	-	-	-	-	-	-	-
AbST	0	0	61-3LV	0	0	0	0	61-3LV	0	0	0	0	0	0	0
Salmochelin	-	-	-	-	-	-	-	-	-	-	-	-	-	-	-
SmST	0	0	0	0	0	0	0	0	0	0	0	0	0	0	0
RmpADC	-	-	-	-	-	-	-	-	-	-	-	-	-	-	-
RmST	0	0	0	0	0	0	0	0	0	0	0	0	0	0	0
rmpA2	-	-	-	-	-	-	-	-	-	-	-	-	-	-	-
wzi	wzi433	wzi2	wzi104	wzi64	wzi24	wzi420	wzi420	wzi104	wzi420	wzi64	wzi64	wzi64	wzi64	wzi2	wzi64
K_locus	KL35	unknown (KL107)	KL51	KL64	KL24	KL10	KL10	unknown (KL107)	KL10	KL64	unknown (KL64)	KL64	unknown (KL64)	KL2	KL64
K_type	K35	unknown (KL107)	K51	K64	K24	K10	K10	unknown (KL107)	K10	K64	unknown (K64)	K64	unknown (K64)	K2	K64
K_locus_problems	none	?-+*	-	?-	?-	none	none	?-+	none	none	-	?-	?-	none	?-
K_locus_confidence	Very high	None	High	Good	Good	Very high	Very high	None	Very high	Perfect	None	Good	None	Very high	Good
K_locus_identity	99.33%	83.35%	99.40%	100.00%	99.99%	96.76%	96.74%	81.68%	96.75%	100.00%	100.00%	100.00%	100.00%	99.73%	99.98%
K_locus_missing_genes		KL107_05_wzb,KL107_06_wzc,KL107_07_wbaP,KL107_08,KL107_09,KL107_10,KL107_11_gnd,KL107_12,KL107_13,KL107_14,KL107_15,KL107_16_ugd	KL51_14_wcoZ	KL64_10_wzy	KL24_13_wcuG			KL107_05_wzb,KL107_06_wzc,KL107_07_wbaP,KL107_08,KL107_09,KL107_10,KL107_11_gnd,KL107_12,KL107_13,KL107_14,KL107_15,KL107_16_ugd			KL64_08_wcoV,KL64_09,KL64_10_wzy,KL64_11_wcoU	KL64_11_wcoU,KL64_12_wcoT	KL64_10_wzy,KL64_11_wcoU,KL64_12_wcoT		KL64_11_wcoU,KL64_12_wcoT
		unknown													

O_locus	O1/O2v1	(OL102) unknown	O1/O2v2	O1/O2v1	O1/O2v1	O3/O3a	O3/O3a	O1/O2v2	O3/O3a	O1/O2v1	O1/O2v1	O1/O2v1	O1/O2v1	O1/O2v1	O1/O2v1
O_type	O1	(OL102)	O1	O2a	O2a	O3/O3a	O3/O3a	O1	O3/O3a	O2a	O2a	O2a	O2a	O1	O2a
O_locus_problems	none	?-	none	?	none	none	none	-	none	none	none	none	none	?-	none
O_locus_confidence	Very high	None	Very high	Good	Very high	Very high	Very high	Good	Very high	Very high	Very high	Very high	Very high	Good	Very high
O_locus_identity	98.17%	76.83%	98.42%	98.64%	98.52%	97.98%	97.98%	98.24%	97.98%	98.64%	98.64%	98.64%	98.64%	99.98%	98.64%
O_locus_missing_genes		OL102_01_rfbB,OL102_02_rfbA,OL102_03_rfbD,OL102_04_rfbC,OL102_05_wbbL,OL102_06_rhamnan,OL102_07_wzm,OL102_08_wzt						O1/O2v2_01_wzm,O1/O2v2_02_wzt						O1/O2v1_06_wbbO	
AGly_acquired	-	-	-	-	strA.v1^;strB.v1	-	-	-	-	strA.v1;strA.v1*;strB.v1;strB.v1*	strA.v1;strA.v1^;strB.v1;strB.v1*	strA.v1;strA.v1^;strB.v1;strB.v1	strA.v1;strA.v1^;strB.v1;strB.v1^	sat-2	strA.v1;strA.v1^;strB.v1;strB.v1
Col_acquired	-	-	-	-	-	-	-	-	-	-	-	-	-	-	-
Fcyn_acquired	-	-	-	-	-	-	-	-	-	-	-	-	-	-	-
Flq_acquired	qnrB1.v2^	-	-	-	-	-	-	-	-	-	-	-	-	-	-
Gly_acquired	-	-	-	-	-	-	-	-	-	-	-	-	-	-	-
MLS_acquired	-	-	-	-	-	-	-	-	-	-	-	-	-	-	-
Phe_acquired	-	-	-	-	-	-	-	-	-	-	-	-	-	-	-
Rif_acquired	-	-	-	-	-	-	-	-	-	-	-	-	-	-	-
Sul_acquired	-	-	-	-	sul2	-	-	-	-	sul2	sul2	sul2	sul2	-	sul2
Tet_acquired	tet(A).v1	-	-	-	tet(A).v1	-	-	-	-	-	-	-	-	-	-
Tgc_acquired	-	-	-	-	-	-	-	-	-	-	-	-	-	-	-
Tmt_acquired	-	-	-	-	-	-	-	-	-	-	-	-	-	dfrA1.v1	-
Bla_acquired	-	-	-	-	-	-	-	-	-	TEM-1D.v1^	TEM-1D.v1^	TEM-1D.v1^	TEM-1D.v1^	-	TEM-1D.v1^
Bla_inhR_acquired	-	-	-	-	-	-	-	-	-	-	-	-	-	-	-
Bla_ESBL_acquired	-	-	CTX-M-15	CTX-M-15	-	CTX-M-15	CTX-M-15	CTX-M-15	CTX-M-15	CTX-M-15	CTX-M-15	CTX-M-15	CTX-M-15	CTX-M-15	CTX-M-15
Bla_ESBL_inhR_acquired	-	-	-	-	-	-	-	-	-	-	-	-	-	-	-
Bla_Carb_acquired	-	-	-	-	NDM-1	-	-	-	-	OXA-181	OXA-181	-	-	OXA-181	-
Bla_chr	SHV-11.v1^	SHV-1^	SHV-11.v1^	SHV-11.v1^	SHV-11.v1	-	-	SHV-1^	-	SHV-11.v1^	SHV-11.v1^	SHV-11.v1^	SHV-11.v1^	SHV-28.v1^	SHV-11.v1^
SHV_mutations	35Q	-	35Q	35Q	35Q	-	-	-	-	35Q	35Q	35Q	35Q	-	35Q
Omp_mutations	-	-	OmpK35-30%;OmpK36TD	OmpK35-76%;OmpK36TD	-	-	-	OmpK35-30%	-	OmpK35-76%;OmpK36TD	OmpK35-76%;OmpK36TD	OmpK35-76%;OmpK36TD	OmpK35-76%;OmpK36TD	OmpK35-66%;OmpK36GD	OmpK35-76%;OmpK36TD
Col_mutations	-	-	-	-	-	-	-	-	-	-	-	-	-	-	-

Flq_mutations	-	-	GyrA-83I;GyrA-87G;ParC-80I	GyrA-83I;ParC-80I	GyrA-83F;GyrA-87A;ParC-80I	GyrA-83Y;GyrA-87A;ParC-80I	GyrA-83Y;GyrA-87A;ParC-80I	GyrA-83I;ParC-80I	GyrA-83Y;GyrA-87A;ParC-80I	GyrA-83I;ParC-80I	GyrA-83I;ParC-80I	GyrA-83I;ParC-80I	GyrA-83I;ParC-80I	GyrA-83Y;GyrA-87G;ParC-80I	GyrA-83I;ParC-80I
truncated_resistance_hits	-	-	-	-	-	-	-	-	-	-	-	-	-	-	-
spurious_resistance_hits	fosA7*?-0%	-	-	-	-	-	-	-	-	-	-	-	-	-	-
Chr_ST	ST1801	ST1634	ST231-1LV	ST147	ST11	ST147	ST147	ST231	ST147	ST147	ST147-1LV	ST147	ST147-1LV	ST14	ST147-1LV
gapA	2	1	2	3	3	3	3	2	3	3	3	3	3	1	3
infB	1	6	6	4	3	4	4	6	4	4	4	4	4	6	4
mdh	1	1	1	6	1	6	6	1	6	6	6	6	6	1	6
pgi	26	26	3	1	1	1	1	3	1	1	1	1	1	1	1
phoE	3	12	26	7	1	7	7	26	7	7	-	7	-	1	-
rpoB	5	25	46	4	1	4	4	1	4	4	4	4	4	1	4
tonB	54	9	77	38	4	38	38	77	38	38	38	38	38	1	38
ybtS	-	23	4	16	65	-	-	4	-	16	16	16*-10%	16*-10%	4	16*-10%
ybtX	-	27	4	12	15	-	-	4	-	12	12	12	12	4	12
ybtQ	-	30	5	4	22	-	-	5	-	4	4	4	4	31	4
ybtP	-	18	4	3	4	-	-	4	-	3	3	3	3	4	3
ybtA	-	13	1	3	1	-	-	1	-	3	3	3	3	1	3
irp2	-	65	8	61	37*	-	-	8	-	61	61	61	61	67	61
irp1	-	61*	8	54	34	-	-	8	-	54	54	54	54	171	54
ybtU	-	9	2	3	14	-	-	2	-	3	3	3	3	2	3
ybtT	-	18	4	10	1	-	-	4	-	10	10	10	10	4	10
ybtE	-	26	4	9	4	-	-	4	-	9	9	9	9	4	9
fyuA	-	35	2	39	17	-	-	2	-	39	39*	39*	39*	36	39*
clbA	-	-	-	-	-	-	-	-	-	-	-	-	-	-	-
clbB	-	-	-	-	-	-	-	-	-	-	-	-	-	-	-
clbC	-	-	-	-	-	-	-	-	-	-	-	-	-	-	-
clbD	-	-	-	-	-	-	-	-	-	-	-	-	-	-	-
clbE	-	-	-	-	-	-	-	-	-	-	-	-	-	-	-
clbF	-	-	-	-	-	-	-	-	-	-	-	-	-	-	-
clbG	-	-	-	-	-	-	-	-	-	-	-	-	-	-	-
clbH	-	-	-	-	-	-	-	-	-	-	-	-	-	-	-
clbI	-	-	-	-	-	-	-	-	-	-	-	-	-	-	-
clbL	-	-	-	-	-	-	-	-	-	-	-	-	-	-	-
clbM	-	-	-	-	-	-	-	-	-	-	-	-	-	-	-
clbN	-	-	-	-	-	-	-	-	-	-	-	-	-	-	-
clbO	-	-	-	-	-	-	-	-	-	-	-	-	-	-	-
clbP	-	-	-	-	-	-	-	-	-	-	-	-	-	-	-
clbQ	-	-	-	-	-	-	-	-	-	-	-	-	-	-	-
iucA	-	-	29	-	-	-	-	29	-	-	-	-	-	-	-
iucB	-	-	17	-	-	-	-	17	-	-	-	-	-	-	-
iucC	-	-	28	-	-	-	-	28	-	-	-	-	-	-	-
iucD	-	-	35	-	-	-	-	35	-	-	-	-	-	-	-
iutA	-	-	61	-	-	-	-	61	-	-	-	-	-	-	-
iroB	-	-	-	-	-	-	-	-	-	-	-	-	-	-	-
iroC	-	-	-	-	-	-	-	-	-	-	-	-	-	-	-
iroD	-	-	-	-	-	-	-	-	-	-	-	-	-	-	-
iroN	-	-	-	-	-	-	-	-	-	-	-	-	-	-	-
rmpA	-	-	-	-	-	-	-	-	-	-	-	-	-	-	-
rmpD	-	-	-	-	-	-	-	-	-	-	-	-	-	-	-
rmpC	-	-	-	-	-	-	-	-	-	-	-	-	-	-	-
spurious_virulence_hits	-	-	-	-	-	-	-	-	-	-	-	-	-	-	-

*Detailed description of the metrics can be found on Kleborate Web-page https://github.com/klebgenomics/Kleborate

Supplementary Table S3. Output of analysis of *K. pneumoniae* plasmids by Mob-Suite software

Metrics*	*Klebsiella pneumoniae* strains														
	CK1	CK2	CK3	CK4	CK5	CK6	CK7	CK8	CK9	K10	K11	K12	K13	K14	K15
num_contigs	1	1	1	1	1	1	1	1	1	1	1	1	1	1	1
size	-	-	-	-	-	-	-	-	-	-	-	-	-	-	-
gc	0.523604645	0.495378342	0.518456727	0.516944998	0.517871413	0.513306639	0.511990677	0.502308814	0.514112032	0.486771719	0.484852804	0.487586348	0.48298567	0.519660243	0.488652363
mpf_type	MPF_F	MPF_F	MPF_F	MPF_I	MPF_F	MPF_F	MPF_F	MPF_F	MPF_F	MPF_I	MPF_I	MPF_I	MPF_I	MPF_F	MPF_I
mpf_type_accession(s)	NC_014312_00116,NC_014312_00118,NC_023332_00154,NC_019389_00068,NC_014312_00128,NC_022609_00081,NC_011281_00082,NC_021654_00016,NC_019165_00034,NC_019155_00032,NC_021502_00131,NC_021502_00150	NC_018107_00101	NC_023332_00154,NC_014312_00118,NC_014312_00116,NC_014312_00128,NC_022609_00081,NC_019389_00080,NC_021654_00016,NC_019165_00034,NC_023905_00125,NC_021502_00131,NC_021502_00150	NC_021198_00249,NC_021198_00243,NC_021198_00240,NC_021198_00239,NC_021198_00238,NC_021198_00236,NC_021198_00235,NC_021198_00234,NC_021198_00230	NC_014312_00116,NC_014312_00118,NC_023332_00154,NC_019389_00068,NC_014312_00128,NC_022609_00081,NC_019389_00080,NC_021654_00016,NC_019165_00034,NC_014312_00141,NC_021198_00249,NC_021198_00243,NC_021198_00240,NC_021198_00239,NC_021198_00238,NC_021198_00236,NC_021198_00235,NC_021198_00234,NC_021198_00230,NC_021502_00131,NC_021502_00150	NC_014312_00116,NC_014312_00118,NC_023332_00154,NC_019389_00068,NC_014312_00128,NC_022609_00081,NC_011281_00082,NC_021654_00016,NC_019165_00034,NC_019155_00032,NC_021502_00131,NC_021502_00150,NC_017624_00009,NC_019083_00030,NC_019157_00030,NC_019157_00029,NC_023277_00163	NC_014312_00116,NC_014312_00118,NC_023332_00154,NC_019389_00068,NC_014312_00128,NC_022609_00081,NC_011281_00082,NC_021654_00016,NC_019165_00034,NC_019155_00032,NC_021502_00131,NC_021502_00150,NC_017624_00009,NC_019083_00030,NC_019157_00030,NC_019157_00029,NC_023277_00163	NC_014312_00116,NC_014312_00118,NC_023332_00154,NC_019389_00068,NC_014312_00128,NC_022609_00081,NC_019389_00080,NC_021654_00016,NC_019165_00034,NC_023905_00125,NC_021079_00038,NC_013718_00045,NC_013718_00044,NC_013718_00041,NC_021502_00131,NC_021502_00150,NC_010726_00011,NC_005814_00013,NC_010726_00014	NC_014312_00116,NC_014312_00118,NC_023332_00154,NC_019389_00068,NC_014312_00128,NC_022609_00081,NC_011281_00082,NC_021654_00016,NC_019165_00034,NC_019155_00032,NC_021502_00131,NC_021502_00150,NC_017624_00009,NC_019083_00030,NC_019157_00030,NC_019157_00029,NC_023277_00163	NC_016980_00110,NC_016980_00109,NC_018107_00235,NC_023911_00309,NC_016980_00240,NC_018107_00101,NC_021198_00249,NC_021198_00243,NC_021198_00240,NC_021198_00239,NC_021198_00238,NC_021198_00236,NC_021198_00235,NC_021198_00234,NC_021198_00230,NC_016980_00099	NC_016980_00110,NC_016980_00109,NC_018107_00235,NC_023911_00309,NC_021198_00249,NC_021198_00243,NC_021198_00240,NC_021198_00239,NC_021198_00238,NC_021198_00236,NC_021198_00235,NC_021198_00234,NC_021198_00230,NC_016980_00099	NC_018107_00101,NC_016980_00240,NC_016980_00110,NC_016980_00109,NC_018107_00235,NC_023911_00309,NC_021198_00249,NC_021198_00243,NC_021198_00240,NC_021198_00239,NC_021198_00238,NC_021198_00236,NC_021198_00235,NC_021198_00234,NC_021198_00230,NC_016980_00099	NC_023911_00309,NC_018107_00235,NC_016980_00109,NC_016980_00110,NC_018107_00101,NC_016980_00240,NC_021198_00249,NC_021198_00243,NC_021198_00240,NC_021198_00239,NC_021198_00238,NC_021198_00236,NC_021198_00235,NC_021198_00234,NC_021198_00230,NC_016980_00099	NC_023332_00154,NC_014312_00118,NC_014312_00116,NC_021231_00085,NC_014312_00128,NC_022609_00081,NC_019389_00080,NC_021654_00016,NC_021654_00004,NC_023905_00125,NC_019165_00034,NC_019155_00032,NC_021502_00131,NC_021502_00150	NC_018107_00101,NC_016980_00240,NC_023911_00309,NC_018107_00235,NC_016980_00109,NC_016980_00110,NC_021198_00249,NC_021198_00243,NC_021198_00240,NC_021198_00239,NC_021198_00238,NC_021198_00236,NC_021198_00235,NC_021198_00234,NC_021198_00230,NC_016980_00099
orit_type(s)	-	-	-	MOBF,MOBP,MOBP,MOB_unknown	MOBF	MOBP,MOB_unknown	MOBP,MOB_unknown	MOBP,MOB_unknown	MOBP,MOB_unknown	MOBF,MOBP,MOBQ,MOBQ,MOB_unknown	MOBF,MOBP,MOBQ,MOBQ,MOB_unknown	MOBF,MOBP,MOBP,MOBQ,MOBQ,MOBQ,MOB_unknown	MOBF,MOBP,MOBP,MOB_unknown	MOBF,MOBP,MOB_unknown	MOBF,MOBP,MOBP,MOB_unknown
orit_accession(s)	-	-	-	KT950740,HQ328804,AP017298,NC_019986	NZ_CP016035	AB027308,CP018359	AB027308,CP018359	AB027308,CP018359	AB027308,CP018359	KT950740,AP017298,NC_013327,NC_009791,NC_019986	KT950740,AP017298,NC_009791,NC_013327,NC_019986	KT950740,HQ328804,AP017298,NC_010672,NC_013327,NC_009791,NC_019986	KT950740,AP017298,HQ328804,NC_019986	NZ_CP016035,AB027308,CP018359	KT950740,HQ328804,AP017298,NC_019986
predicted_mobility	conjugative	conjugative	conjugative	conjugative	conjugative	conjugative	conjugative	conjugative	conjugative	conjugative	conjugative	conjugative	conjugative	conjugative	conjugative
mash_nearest_neighbor	CP025457	CP019161	MK649824	CP024839	CP022125	CP024500	CP024500	MK649824	CP024500	CP034040	HG918041	CP034040	CP034040	CP028930	CP034040
mash_neighbor_distance	0.00940624	0.0229306	0.0106545	0.0282247	0.016782	0.0124	0.0158335	0.0188675	0.0143027	0.0335842	0.0396325	0.035505	0.0368186	0.0237537	0.0352724
mash_neighbor_identification	Klebsiella pneumoniae	Klebsiella pneumoniae	Klebsiella pneumoniae	Klebsiella pneumoniae	Klebsiella pneumoniae	Klebsiella pneumoniae	Klebsiella pneumoniae	Klebsiella pneumoniae	Klebsiella pneumoniae	Klebsiella pneumoniae subsp. pneumoniae	Klebsiella pneumoniae	Klebsiella pneumoniae subsp. pneumoniae	Klebsiella pneumoniae subsp. pneumoniae	Klebsiella pneumoniae	Klebsiella pneumoniae subsp. pneumoniae
primary_cluster_id	AA276	AA397	AA021	AA964	AA275	AA277	AA277	AA021	AA277	AA405	AA405	AA405	AA405	AA457	AA405
secondary_cluster_id	AI081	AI419	AH570	AJ456	AI078	AI089	AI089	AH570	AI089	AI437	AI443	AI437	AI437	AI561	AI437
predicted_host_range_overall_rank	order	order	multi-phylla	multi-phylla	order	multi-phylla	multi-phylla	multi-phylla	multi-phylla	class	multi-phylla	multi-phylla	multi-phylla	order	multi-phylla
predicted_host_range_overall_name	Enterobacterales	Enterobacterales	Actinomycetota,Pseudomonadota	Actinomycetota,Bacillota,Bacteroidota,Pseudomonadota	Enterobacterales	Actinomycetota,Pseudomonadota	Actinomycetota,Pseudomonadota	Actinomycetota,Pseudomonadota	Actinomycetota,Pseudomonadota	Gammaproteobacteria	Actinomycetota,Bacillota,Bacteroidota,Pseudomonadota	Actinomycetota,Bacillota,Bacteroidota,Pseudomonadota	Actinomycetota,Bacillota,Bacteroidota,Pseudomonadota	Enterobacterales	Actinomycetota,Bacillota,Pseudomonadota
observed_host_range_ncbi_rank	order	order	multi-phylla	multi-phylla	order	multi-phylla	multi-phylla	multi-phylla	multi-phylla	class	multi-phylla	multi-phylla	multi-phylla	order	multi-phylla
observed_host_range_ncbi_name	Enterobacterales	Enterobacterales	Actinomycetota,Pseudomonadota	Actinomycetota,Bacillota,Bacteroidota,Pseudomonadota	Enterobacterales	Actinomycetota,Pseudomonadota	Actinomycetota,Pseudomonadota	Actinomycetota,Pseudomonadota	Actinomycetota,Pseudomonadota	Gammaproteobacteria	Actinomycetota,Bacillota,Bacteroidota,Pseudomonadota	Actinomycetota,Bacillota,Bacteroidota,Pseudomonadota	Actinomycetota,Bacillota,Bacteroidota,Pseudomonadota	Enterobacterales	Actinomycetota,Bacillota,Pseudomonadota
reported_host_range_lit_rank	order	order	order	order	order	order	order	order	order	order	order	order	order	order	order

reported_host_range_lit_name	Enterobacterales	Enterobacterales	Enterobacterales	Enterobacterales	Enterobacterales	Enterobacterales	Enterobacterales	Enterobacterales	Enterobacterales	Enterobacterales	Enterobacterales	Enterobacterales	Enterobacterales	Enterobacterales	Enterobacterales
associated_pmid(s)	11254626; 19482926; 15781495; 20851899; 16257053	18606786; 11254626; 19482926; 15781495; 20851899; 16257053	10773089; 11254626; 25389419; 20547789; 19482926; 19897651; 26169555; 15781495; 24247128; 20851899; 22511964; 16257053	17122402; 16940067; 6263753; 20008783; 23711894; 26802429	10773089; 11254626; 16940067; 25389419; 20547789; 19482926; 19897651; 23711894; 15781495; 24247128; 20851899; 22511964; 16257053	11254626; 30761100; 19482926; 15781495; 26842776; 20851899; 16257053; 22470007	11254626; 30761100; 19482926; 15781495; 26842776; 20851899; 16257053; 22470007	10773089; 11254626; 25389419; 30761100; 28336940; 19482926; 20547789; 21911569; 19897651; 24247128; 15781495; 26842776; 20851899; 22511964; 16257053	11254626; 30761100; 19482926; 15781495; 26842776; 20851899; 16257053; 22470007	18606786; 11254626; 16940067; 19482926; 23711894; 15781495; 20851899; 16257053	18606786; 11254626; 16940067; 17122402; 6263753; 19482926; 16257053; 23711894; 15781495; 20851899; 26802429	18606786; 11254626; 16940067; 17122402; 6263753; 19482926; 16257053; 23711894; 15781495; 20851899; 26802429	18606786; 11254626; 16940067; 17122402; 6263753; 19482926; 20008783; 16257053; 23711894; 15781495; 20851899; 26802429	10773089; 11254626; 25389419; 30761100; 19482926; 19897651; 23711894; 15781495; 24247128; 20851899; 22511964; 16257053	18606786; 11254626; 16940067; 19482926; 20008783; 23711894; 15781495; 20851899; 16257053

*Detailed description of the metrics can be found on Mob-Suite Web-page https://github.com/phac-nml/mob-suite

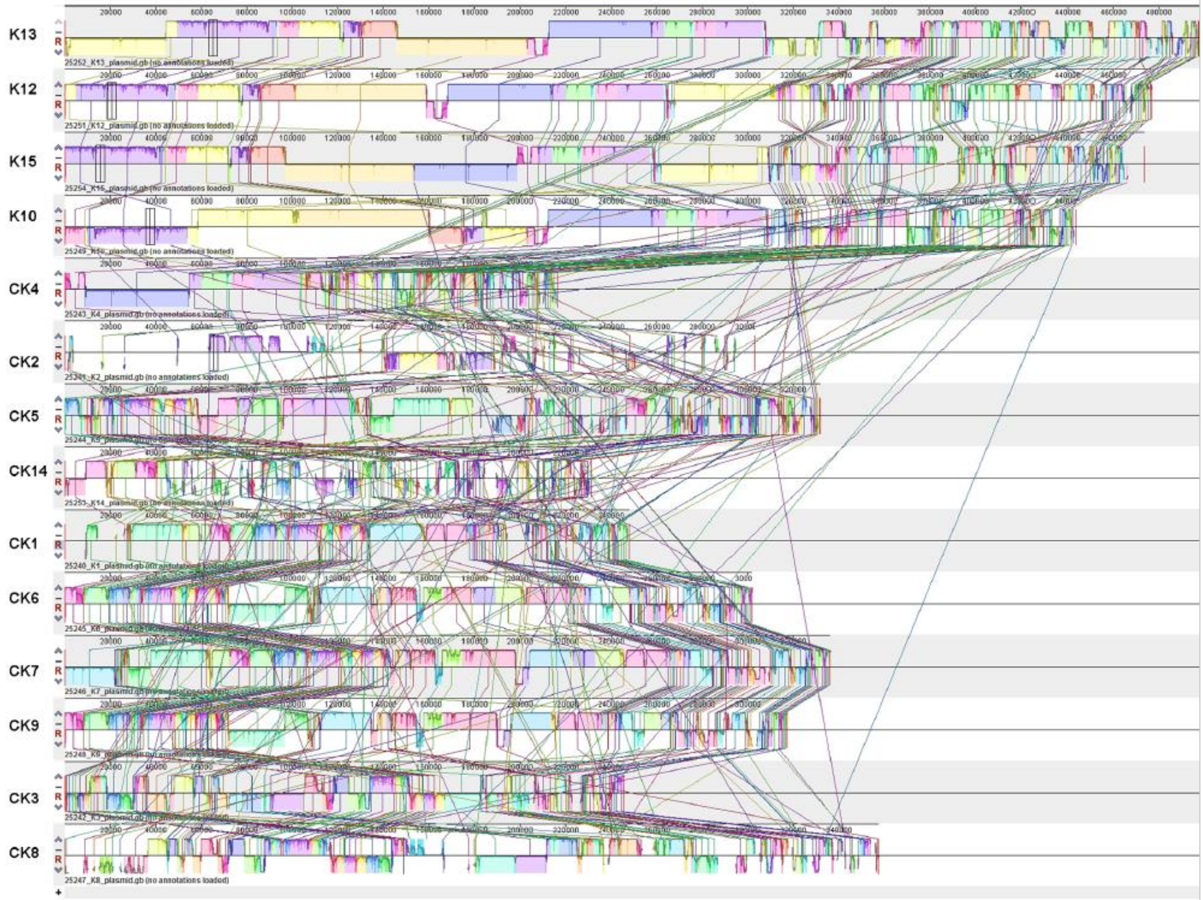

Supplementary Figure S1. Alignment of sequences of the plasmids from different strains of *K. pneumoniae* produced by Mauve 20150226.
Each sequence is represented by a row with a central line and sequence length values. Blocks filled with the same colour and connected by thin vertical lines represent homologous sequences found in different plasmids. Blocks above the central line are located on the forward strand and those below the line are located on the reverse complement strand of the plasmids.
